# Body Weight Variability Increases Dementia Risk Among Older Adults: A Nationwide Population-Based Cohort Study

**DOI:** 10.3389/fendo.2020.00291

**Published:** 2020-05-12

**Authors:** Eun Roh, Soon Young Hwang, Jung A. Kim, You-Bin Lee, So-hyeon Hong, Nam Hoon Kim, Ji A. Seo, Sin Gon Kim, Nan Hee Kim, Kyung Mook Choi, Sei Hyun Baik, Hye Jin Yoo

**Affiliations:** ^1^Division of Endocrinology and Metabolism, Department of Internal Medicine, Korea University College of Medicine, Seoul, South Korea; ^2^Department of Biostatistics, Korea University College of Medicine, Seoul, South Korea

**Keywords:** body weight, variability, dementia, Alzheimer's dementia, older people, retrospective cohort study

## Abstract

**Background:** Recent growing evidences suggest that body weight (Bwt) variability, a repeated loss and regain of weight within a specific period, causes metabolic disturbances and can be a marker for poor homeostasis. Although there have been many studies about the association between Bwt variability and various health status, its association with the incidence of dementia among elderly people has not been examined.

**Methods:** We performed a retrospective elderly cohort study from 19,987 participants with mean age 73 years old in the Korean National Health Insurance Service. We examined the risk of incident dementia, including Alzheimer's dementia and vascular dementia, according to the quartile of Bwt variability, represented as coefficient of variation (Bwt-CV), SD (Bwt-SD), and variability independent of the mean (Bwt-VIM).

**Results:** In fully adjusted model, the group with the highest Bwt variability (Bwt-VIM Q4) showed an increased risk of all-cause dementia (hazard ratio [HR] 1.39, 95% confidence interval [CI] 1.206–1.603) and Alzheimer's dementia (HR 1.46, CI 1.240–1.724) compared to the lowest quartile (Bwt-VIM Q1). We also found that subjects with the highest Bwt variability (Q4) and underweight BMI had a significantly increased risk of developing dementia (HR 1.994, 95% CI 1.302–3.054), while subjects with low Bwt variability (Q1 and Q2) and obese BMI had decreased risk of dementia (HR 0.664, 95% CI 0.505–0.872 and HR 0.648, 95% CI 0.493–0.852, respectively) compared to reference group (lowest Bwt variability (Q1) with normal baseline BMI). The effect of Bwt variability on the incidence of dementia was more prominent in subjects <75 years old and abnormal BMI groups (P for interaction < 0.05).

**Conclusion:** The present study revealed that high Bwt variability was associated with an increased risk of dementia in the elderly.

## Introduction

The worldwide epidemic of dementia is a major health problem with significant economic consequences. The number of patients with dementia has increased worldwide because of a growing population of older adults. Several risk factors for the development of dementia have been identified. Although increasing age is well-accepted as a risk factor for the development of dementia, dementia is not regarded as an inevitable consequence of aging (1). Dementia shares many modifiable risk factors, including high blood pressure, obesity, physical inactivity, and unhealthy diet, with other major chronic late-life disorders, such as ischemic heart disease and cerebrovascular disease (1). On the bases of epidemiological and clinical evidence, mid-life obesity has been identified as a risk factor for late-life dementia through the direct effect of adipocyte-derived hormones and cytokines on cognitive function as well as the indirect impact on vascular risk factors (2–6). In contrast, in late-life, a low body mass index (BMI) or being underweight late in life was a risk factor for dementia and age-related brain atrophy in several prospective and cross-sectional studies (5, 7–9). However, these studies require attention to interpretation because a low BMI in the elderly might result from decreased muscle mass, not fat mass (10), and weight loss is a potential preclinical marker for dementia 6–10 years before a clinical diagnosis (11). Therefore, body weight (Bwt) trajectory rather than Bwt at a specific time might provide more information on the risk of dementia late in life.

Recent growing evidences suggest that variability in metabolic parameters may have a role in predicting mortality and cardiovascular outcomes (12–17). A loss of physiological homeostasis in the elderly can lead to intrinsic variability in various physiological indices, ultimately impairing health or causing disease. Under physiological conditions, homeostatic adaptations protect against weight gain or loss (18). Bwt variability, same meaning as Bwt fluctuation or weight cycling, refers to the repeated loss and regain of weight within a specific period. A meta-analysis of 25 studies involving more than 400,000 participants, Bwt fluctuation was associated with a significant increase in risk of all-cause mortality, cardiovascular disease (CVD) mortality, and CVD (17). Although there have been some studies to examine the association between BMI at a specific time or Bwt changes with dementia (19–21), there has been no study to explore the effects of Bwt variability in late-life on the incidence of dementia.

Thus, to clarify the importance of healthy Bwt control in older people, which can help maintain cognitive function, the present study investigated the association between visit-to-visit Bwt variability and the risk for all-cause dementia and dementia subtypes in an elderly population by using the longitudinal National Health Insurance Service (NHIS)-Senior database. Because baseline BMI can influence the risk of dementia in the elderly, we also analyzed the association between Bwt variability and dementia according to baseline BMI.

## Materials and Methods

### Study Population

The NHIS is a mandatory social health insurance program that enrolls about 98% of Koreans who participate in biannual standardized health examinations provided by the Korean government (22, 23). The NHIS-Senior database was used to randomly select ~10% of NHIS participants who were aged more than 60 years. The NHIS-Senior database contains an eligibility database (including data such as age, sex, and socioeconomic variables), a health examination database (comprising questionnaires on health-related behavioral variables and results of laboratory measurements), and a medical history database (comprising information on diagnosis, medication, admission, and death). Quality control procedures were checked by the Korean Association of Laboratory Quality Control.

**Figure 1:**
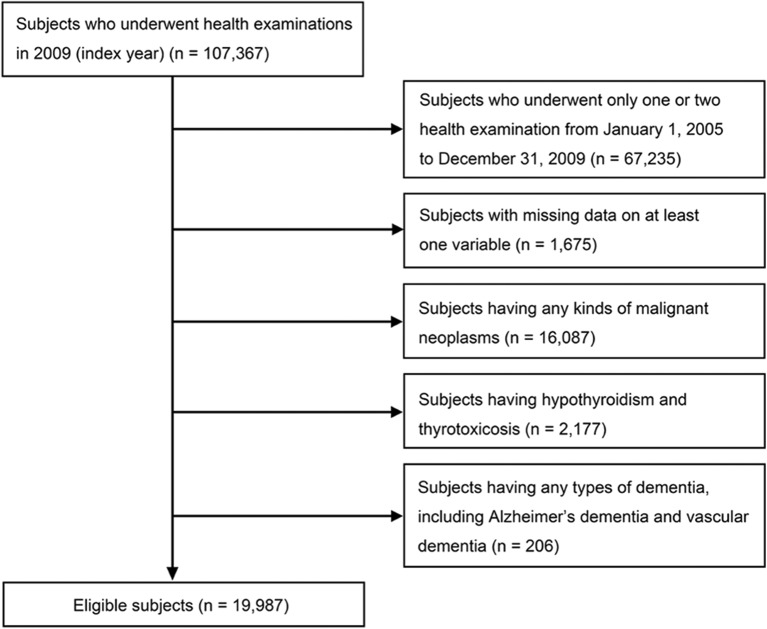
Flowchart of the study population.

Data from participants who underwent the national health examinations in 2009 (index year), and three or more health examinations from January 1, 2005, to December 31, 2009 were included. A total of 67,235 participants who underwent only one or two examinations and 1,675 participants with missing on at least one variable were excluded. An additional 206 participants were excluded due to a previous diagnosis of any type of dementia, including Alzheimer's dementia and vascular dementia, based on diagnoses coded according to the International Classification of Diseases, 10th revision (ICD-10) and questionnaires about medical history before the index year. Subjects who had diseases that could change Bwt, such as any kinds of malignant neoplasm (C00–C97; *n* = 16,087), hypothyroidism or thyrotoxicosis (E03, E05; *n* = 2,177), were excluded. A total 19,987 individuals were finally included in the analysis (Figure 1). The protocols were approved by the NHIS review committee, and the Institutional Review Board of Korea University approved the study protocol in accordance with the Declaration of Helsinki of the World Medical Association (IRB No. K2018–1107-001). Informed consent was waived because anonymous and de-identified information was used for analysis.

### Measurements and Definitions

Anthropometric and laboratory measurements were performed after an overnight fast. Body mass index (BMI) was calculated as Bwt in kilograms (kg) divided by the square of height in meters (m^2^). Subjects were categorized into 4 groups according to the World Health Organization recommendation for Asian population: <18.5 kg/m^2^ (underweight), 18.5–22.9 kg/m^2^ (normal), 23.0–24.9 kg/m^2^ (overweight), and BMI ≥25 kg/m^2^ (obese) (19). Information on current smoking status, alcohol consumption (at least 1 drink per week), and regular exercise (strenuous physical activity on at least 3 times per week) were obtained from a questionnaire filled-in during the health examination. Income levels were dichotomized at the lower 10%.

Diabetes was defined as fasting glucose level ≥126 mg/dL or at least one prescription claim per year for antidiabetic medication under the International Classification of Diseases, 10th revision (ICD-10) codes (E10–E14). Hypertension was defined as systolic/diastolic blood pressure ≥140/90 mmHg or at least one prescription claim per year for an antihypertensive agent under ICD-10 codes (I10–I13, I15). Dyslipidemia was defined as total cholesterol levels ≥240 mg/dL or at least one prescription claim per year for an antihyperlipidemic agent under ICD-10 codes (E78). Ischemic heart disease (I20–25) and cerebrovascular disease (I60–69) were assessed by searching for ICD-10 codes.

### Definition of Bwt Variability

Visit-to-visit Bwt variability was determined from at least three Bwt measurements for each participant. A total of 17,681 participants (88.5%) had three measurements, 1,548 participants (7.8%) had four measurements, and 758 participants (3.8%) had five measurements. We measured intra-individual Bwt variability using three indices of variability, namely variability independent of the mean (Bwt-VIM), coefficient of variation (Bwt-CV), and standard deviation (Bwt-SD). Bwt-SD can increase as the mean Bwt level increases. CV was defined as (SD/mean) × 100 (%) to exclude the effect of mean Bwt level, but it had concern that it does not completely rule out the effects of mean Bwt level. Bwt-VIM is another visit-to-visit Bwt variability measurement that has no correlation with mean Bwt level over visits. VIM was (k × SD(Bwt))/mean(Bwt)^x^, where x is calculated from fitting a power model: SD (Bwt) = a times mean(Bwt)^x^ and k = mean(mean(Bwt))^x^ (24, 25).

### Outcomes

We examined new cases of dementia in the governmental database from January 1, 2009 to December 31, 2015. The incidence of all-cause dementia was determined through prescriptions for an anti-dementia drug (donepezil, galantamine, rivastigmine, or memantine), defined as ICD-10 codes (F00, F01, F02, F03, and G30) used as the first or second diagnosis for medical expense claims submitted to the NHIS until the end of follow-up. Alzheimer's dementia was specified by ICD-10 codes F00 or G30, while vascular dementia was defined by ICD-10 code F01. When both Alzheimer's disease and vascular dementia codes were used, the final diagnosis was defined as vascular dementia. This definition was applied to both outpatients and hospitalized patients. To submit a valid claim for an anti-dementia drug prescription, physicians need to document evidence of cognitive decline according to relatively strict criteria: (1) Mini Mental State Examination score ≤ 26 and (2) either a Clinical Dementia Rating ≥ 1 or a Global Deterioration Scale score ≥ 3 (26).

### Statistical Analysis

Data were analyzed with SAS 9.4 (SAS Institute Inc., Cary, NC, USA). Results are presented as the median (interquartile range) for continuous variables and counts (percentage, %) for categorical variables. Normal distribution was determined by Kolmogorov-Smirnov test. Statistical analysis for between-group comparisons was based on Kruskal-Wallis test or Mann-Whitney test for continuous variables or χ^2^ test for categorical variables. Kaplan–Meier curves for cumulative incidence of dementia and dementia subgroups were obtained for four groups, classified as according to quartile of Bwt variability. Cox proportional hazards models were used to calculate hazard ratios (HRs) and 95% confidence intervals (CIs) for the risk of dementia by quartile of Bwt variability after adjusting for confounding factors including age, sex, BMI, alcohol consumption, current smoking, exercise, income, diabetes mellitus, hypertension, dyslipidemia, heart failure, ischemic heart disease, and cerebrovascular disease. HRs (95% CIs) for the incidence of dementia was also calculated according to quartiles of Bwt variability and four categories of baseline BMI with interaction effect after adjusting for multiple confounding factors. Subgroup analysis was conducted by stratifying for age, sex, BMI, current smoking status, alcohol consumption, regular exercise, income, diabetes, hypertension, dyslipidemia, ischemic heart disease, and cerebrovascular disease. In subgroup analyses, the HR and 95% CI of the highest quartile (Q4) group were compared with those of the lower three quartiles (Q1–Q3) as the reference group by using Cox proportional hazards regression models with interaction effect. Sensitivity analyses were also performed, excluding subjects over 85 years of age, or subjects with continuous weight loss or gain. Statistical significance was assumed at *P* <0.05. All statistical analyses were performed by an experienced professional statistician, who was also one of authors.

## Results

### Baseline Characteristics and Incidence of Dementia

Baseline characteristics of the study subjects according to the presence of dementia are presented in Supplementary Table 1. The mean age of our study population in 2009 (index year) was 73.1 years (*SD* = 4.1, range 67–98). Subjects with dementia were older and more likely to be female when compared to subjects without dementia. Individuals with dementia had lower BMI, Bwt, and waist circumference and a higher Bwt variability. The percentages of current smokers and alcohol consumers were lower in dementia group. They also had a higher prevalence of diabetes, dyslipidemia, cerebrovascular disease, and ischemic heart disease.

Table 1 shows the baseline characteristics of study subjects according to Bwt-VIM quartile. The mean values of anthropometric measurements, BMI, proportions of lifestyle characteristics, and the prevalence of diabetes, hypertension, and cerebrovascular disease differed significantly among the Bwt-VIM quartile. During the median follow-up period of 6.5 years (range, 6.2–6.8 years), 1,592 (7.97%) of the 19,987 subjects with complete follow-up data had all-cause dementia, of which 1,217 (6.09%) had Alzheimer's dementia and 304 (1.52%) had vascular dementia. The ratio of Alzheimer's dementia to vascular dementia was 4.0, similar to previous Korean prevalence studies (27).

**Table 1:** Baseline characteristics of the subjects according to the quartiles of body weight (Bwt) variability.

	**Q1** ***N* = 4,996**	**Q2** ***N* = 4,985**	**Q3** ***N* = 5,011**	**Q4** ***N* = 4,995**	***P*-value**
Age (years)	72 (70, 74)	72 (70, 75)	72 (70, 76)	72 (70, 76)	<0.001
Sex (male) (*n*, %)	3,131 (62.7)	2,946 (59.1)	2,940 (58.7)	3,008 (60.2)	<0.001
BMI (kg/m^2^)	23.85 (22.05, 25.56)	23.53 (21.72, 25.46)	23.18 (21.17, 25.28)	23.39 (21.22, 25.51)	<0.001
Body weight (kg)	60 (55, 66)	59 (53, 66)	58 (50, 65)	59 (52, 66)	<0.001
Waist circumference (cm)	84 (79, 89)	83 (78, 88)	82 (76, 88)	83 (78, 90)	<0.001
Body weight variability	0.59 (0.57, 0.97)	1.21 (1.13, 1.5)	2 (1.73, 2.11)	3.13 (2.67, 4.01)	<0.001
VIM	0.59 (0.57, 0.97)	1.21 (1.13, 1.5)	2 (1.73, 2.11)	3.13 (2.67, 4.01)	<0.001
CV (%)	1.08 (0.89, 1.47)	2.17 (1.92, 2.45)	3.3 (2.96, 3.7)	5.41 (4.54, 6.93)	<0.001
SD (mg/dL)	0.58 (0.58, 1)	1.15 (1.15, 1.53)	2 (1.73, 2.08)	3.06 (2.65, 4.04)	<0.001
Incidence of dementia (*n*, %)					
All-cause dementia	318 (6.4)	351 (7.0)	432 (8.6)	491 (9.8)	<0.001
Vascular dementia	69 (1.4)	67 (1.3)	74 (1.5)	94 (1.9)	0.035
Alzheimer's dementia	232 (4.6)	268 (5.4)	339 (6.8)	378 (7.6)	<0.001
Systolic BP (mmHg)	130 (120, 140)	130 (120, 140)	130 (120, 139)	130 (120, 140)	0.059
Diastolic BP (mmHg)	80 (70, 83)	80 (70, 84)	80 (70, 84)	80 (70, 85)	0.322
Fasting plasma glucose (mg/dL)	97 (89, 109)	97 (89, 108)	96 (88, 108)	97 (89, 109)	0.001
Total cholesterol (mg/dL)	194 (172, 220)	195 (172, 220)	195 (171, 221)	193 (169, 219)	0.001
Aspartate transaminase (IU/L)	24 (20, 29)	24 (20, 29)	24 (20, 29)	24 (20, 30)	0.180
Alanine transaminase (IU/L)	19 (15, 25)	19 (15, 25)	19 (15, 25)	19 (15, 26)	0.009
Current smoker (*n*, %)	731 (14.6)	705 (14.1)	774 (15.5)	778 (15.6)	0.141
Alcohol consumption (*n*, %)	1,676 (33.6)	1,584 (31.8)	1,609 (32.1)	1,530 (30.6)	0.019
Regular exercise (*n*, %)	1,113 (22.3)	978 (19.6)	944 (18.8)	900 (18)	<0.001
Diabetes (*n*, %)	1,229 (24.6)	1,199 (24.1)	1,200 (24)	1,330 (26.6)	0.006
Hypertension (*n*, %)	3,107 (62.2)	3,182 (63.8)	3,177 (63.4)	3,324 (66.6)	<0.001
Dyslipidemia (*n*, %)	1,686 (33.8)	1,639 (32.9)	1,657 (33.1)	1,643 (32.9)	0.769
erebrovascular disease (*n*, %)	412 (8.3)	402 (8.1)	456 (9.1)	492 (9.9)	0.005
Ischemic heart disease (*n*, %)	547 (11)	584 (11.7)	584 (11.7)	610 (12.2)	0.268
Income (lower 10%)	547 (11)	565 (11.3)	550 (11)	569 (11.4)	0.845

*P-value derived using Kruskal–Wallis test and χ^2^ test*.

*Data are expressed as median (interquartile range), or n (%)*.

*BMI, body mass index; BP, blood pressure; CV, coefficients of variance; SD, standard deviation; VIM, variability independent of the mean*.

### Effects of Bwt Variability on Incident Dementia

**Figure 2:**
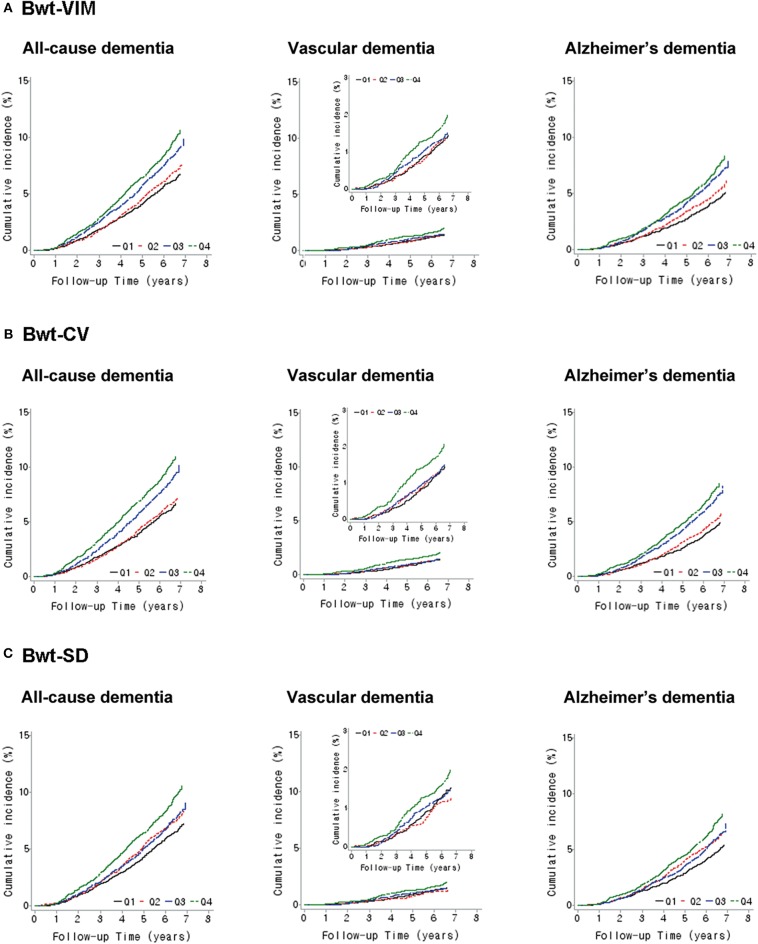
Kaplan–Meier estimates of cumulative incidence of dementia according to quartiles of body weight (Bwt) variability **(A)** Bwt-VIM, **(B)** Bwt-CV, **(C)** Bwt-SD. CV, coefficients of variance; SD, standard deviation; VIM, variability independent of the mean.

A Kaplan-Meier plot was used to describe the cumulative incidence of dementia according to Bwt-VIM quartile (Figure 2A). The incidence of all-cause dementia and Alzheimer's dementia increased as Bwt variability increased. The annual incidence of all-cause dementia was 10.00 per 1,000 person-years in Bwt-VIM Q1 and 15.66 per 1,000 person-years in Bwt-VIM Q4. In Alzheimer's dementia, the annual incidence was 7.24 per 1,000 person-years in Bwt-VIM Q1 and 11.92 per 1,000 person-years in Bwt-VIM Q4 group. The risk of all-cause dementia and Alzheimer's dementia were highest in Bwt-VIM Q4 (log rank test, *P* < 0.001, both). These results were consistent when Bwt variability was determined with CV or SD (Figures 2B,C).

Table 2 indicates that the association between Bwt variability and the incidence of all-cause dementia and Alzheimer's dementia remained after adjusting for baseline covariates. Subjects in the Bwt-VIM Q4 had a higher risk of all-cause dementia (unadjusted, HR 1.570, 95% CI 1.363–1.808) than did subjects in the Bwt-VIM Q1. After adjusting for age, sex, BMI, alcohol consumption, smoking, exercise, income, diabetes, hypertension, dyslipidemia, ischemic heart disease, and cerebrovascular disease (Model 3), the association persisted (HR 1.390, 95% CI 1.206–1.603 for Q4 compared to Q1). The association between Bwt variability and risk of Alzheimer's dementia was especially significant. The fully adjusted HRs for the incidence of Alzheimer's dementia for the Bwt-VIM Q4 vs. Bwt-VIM Q1 was 1.462 (95% CI 1.240–1.724). For vascular dementia, the association between Bwt variability and the risk of dementia was attenuated in the adjusted models (Model 3, HR 1.204, 95% CI 0.880–1.647 for Q4 compared to Q1).

**Table 2:** Hazard ratios and 95% confidence intervals of incidence dementia by quartiles of body weight (Bwt) variability.

	**Total (n)**	**Events (n)**	**Follow-up duration (person years)**	**Incidence rate (per 1,000 person years)**	**Hazard ratio (95% confidence intervals)**			
					**Unadjusted**	**Model 1**	**Model 2**	**Model 3**
**All-cause dementia**								
Q1	4,996	318	31791.3	10.00	1 (ref)	1 (ref)	1 (ref)	1 (ref)
Q2	4,985	351	31702.84	11.07	1.106 (0.951, 1.288)	1.079 (0.927, 1.256)	1.070 (0.919, 1.245)	1.074 (0.923, 1.250)
Q3	5,011	432	31607.93	13.67	1.369 (1.185, 1.583)	1.294 (1.119, 1.495)	1.268 (1.097, 1.467)	1.265 (1.094, 1.464)
Q4	4,995	491	31351.62	15.66	1.570 (1.363, 1.808)	1.421 (1.233, 1.637)	1.403 (1.217, 1.617)	1.390 (1.206, 1.603)
*P* for trend					<0.001	<0.001	<0.001	<0.001
**Vascular dementia**								
Q1	4,996	69	32433.34	2.13	1 (ref)	1 (ref)	1 (ref)	1 (ref)
Q2	4,985	67	32399.7	2.07	0.971 (0.694, 1.360)	0.953 (0.681, 1.334)	0.944 (0.674, 1.322)	0.945 (0.675, 1.324)
Q3	5,011	74	32523.6	2.28	1.070 (0.771, 1.486)	1.025 (0.738, 1.424)	1.004 (0.722, 1.395)	0.982 (0.706, 1.366)
Q4	4,995	94	32354.95	2.91	1.368 (1.002, 1.866)	1.268 (0.928, 1.732)	1.251 (0.915, 1.710)	1.204 (0.880, 1.647)
*P* for trend					0.040	0.122	0.149	0.242
**Alzheimer's dementia**								
Q1	4,996	232	32053.83	7.24	1 (ref)	1 (ref)	1 (ref)	1 (ref)
Q2	4,985	268	31934.62	8.39	1.159 (0.972, 1.381)	1.129 (0.947, 1.346)	1.118 (0.938, 1.333)	1.124 (0.943, 1.341)
Q3	5,011	339	31909.59	10.62	1.471 (1.245, 1.738)	1.386 (1.173,1.638)	1.357 (1.147, 1.605)	1.362 (1.151, 1.611)
Q4	4,995	378	31724.19	11.92	1.649 (1.400, 1.941)	1.483 (1.258, 1.748)	1.463 (1.241, 1.725)	1.462 (1.240, 1.724)
*P* for trend					<0.001	<0.001	<0.001	<0.001

*Model 1: Adjusted for age, sex*.

*Model 2: model 1 + BMI*.

*Model 3: model 2 + alcohol, smoking, exercise, income, diabetes, hypertension, dyslipidemia, ischemic heart disease, cerebrovascular disease*.

### Effects of Baseline BMI and Bwt Variability on the Incidence of Dementia

We analyzed the baseline characteristics of study subjects according to baseline BMI (Supplementary Table 2). Underweight subjects had the highest Bwt variability indices and highest prevalence of all-cause dementia, Alzheimer's dementia, and vascular dementia. Subjects with a lower baseline BMI were more likely to be current smokers and less likely to exercise regularly. In contrast, obese subjects had the highest mean of Bwt, waist circumference, fasting glucose and total cholesterol, and prevalence of diabetes, hypertension, dyslipidemia and ischemic heart disease.

**Figure 3:**
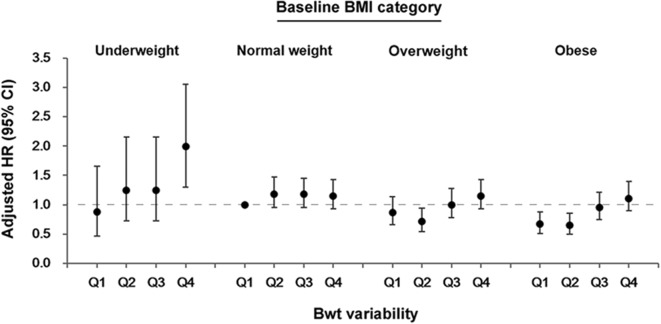
Association between body weight (Bwt) variability and the risk of dementia according to baseline body mass index (BMI) category. CI, confidence intervals; HR, hazard ratios.

The association between Bwt variability and dementia differed according to baseline BMI category (Figure 3 and Supplementary Table 3, *P*-value for interaction between Bwt variability and baseline BMI category = 0.034). To analyze the effect of Bwt variability on the risk of dementia according to baseline BMI, we performed analyses using a single reference group (normal baseline BMI with Bwt-VIM Q1). Subjects in underweight BMI with Bwt-VIM Q4 group had a significantly increased risk of developing dementia (HR 1.994, 95% CI 1.302–3.054). Subjects in obese BMI with Bwt-VIM Q1 and Q2 groups had a decreased risk of developing dementia (HR 0.664, 95% CI 0.505–0.872 and HR 0.648, 95% CI 0.493–0.852, respectively).

### Subgroup Analysis

**Figure 4:**
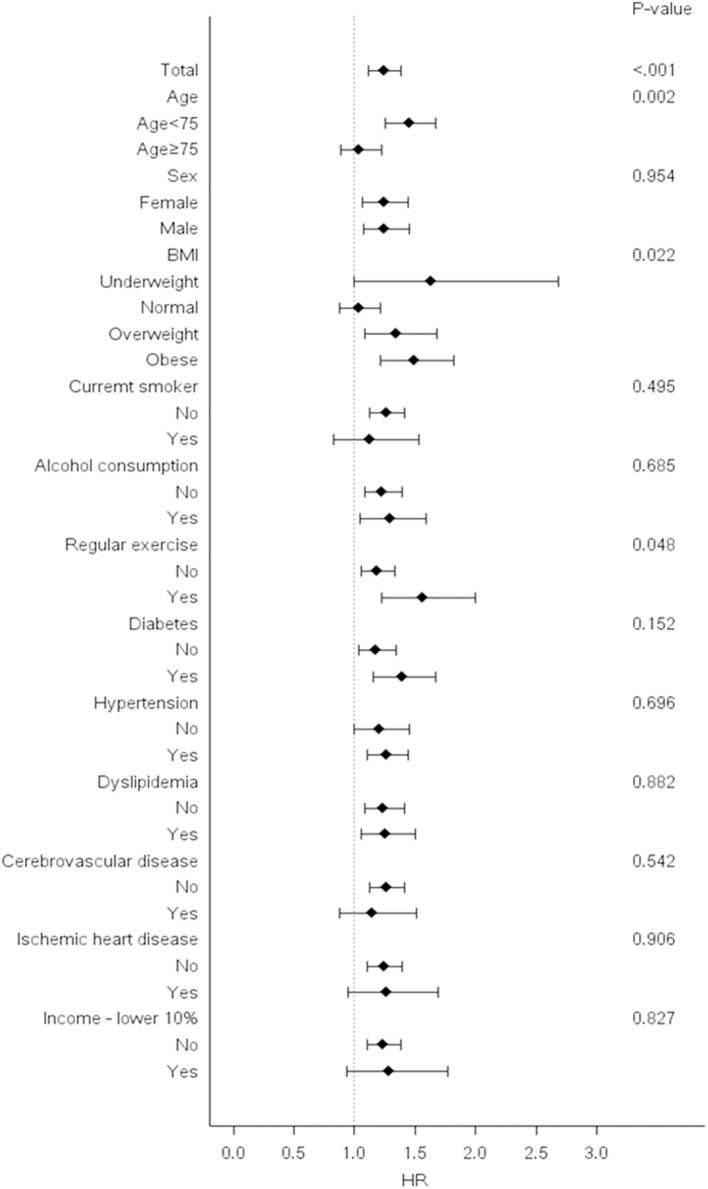
Risk of dementia in the highest vs. lower three quartiles of body weight (Bwt) variability in subgroups. Hazard ratios and 95% confidence intervals for the risk of dementia in the highest quartile (Q4) vs. lower three quartiles (Q1–Q3) of Bwt variability in subgroups. BMI, body mass index; HR, hazard ratios.

Stratified analyses were conducted by age, sex, smoking status, alcohol consumption, income and presence or absence of diabetes, hypertension, dyslipidemia, cerebrovascular disease, and ischemic heart disease. Bwt-VIM Q4 remained predictive of dementia in almost all subgroups when compared with Q1–Q3. In subgroup analysis, higher adjusted HRs of all-cause dementia were observed among subjects with younger age (age <75), subjects with abnormal BMI (underweight, overweight, and obese), and subjects who participated in regular exercise (Figure 4).

### Sensitivity Analysis

The impact of body weight variability on the risk of dementia is very likely to be influenced by age. To exclude the possible effects of prodromal symptoms of Alzheimer's disease in oldest-old, an additional analysis was performed except for those over 85 years of age (Supplementary Table 4). The fully adjusted HRs for the incidence of all-cause dementia and Alzheimer's dementia for the Bwt-VIM Q4 vs. Bwt-VIM Q1 were 1.426 (95% CI 1.236–1.646) and 1.513 (95% CI 1.281–1.787), respectively. Bwt variability means intra-individual weight cycling within specific period, and it is different concept with the simple weight loss and weight gain. Thus, we conducted further analysis after excluding subjects with continuous weight loss or gain (Supplementary Table 5). Subjects in the Bwt-VIM Q4 had higher risks of all-cause dementia (HR 1.325, 95% CI 1.127–1.557) and Alzheimer's dementia (HR 1.352, 95% CI 1.122–1.63) than did subjects in the Bwt-VIM Q1 in Model 3.

## Discussion

The present study provides the first demonstration that high Bwt variability in the elderly was significantly associated with the risk of all-cause dementia and Alzheimer's dementia after extensive adjustment for possible covariates in the Korean population. This association remained statistical significant even after excluding the subjects with continuous weight low or gain. Furthermore, in individuals with an obese BMI at baseline, lower Bwt variability was associated with decreased risk of dementia, whereas the subjects with underweight BMI and the highest Bwt variability showed a significantly increased risk of developing dementia.

Epidemiologic studies have shown that higher adiposity at mid-life, whether measured as BMI or central obesity, was associated with a higher risk of dementia (3, 5, 6). The mechanisms linking obesity with cognitive decline and dementia have been demonstrated in several studies. In middle aged adults, visceral fat volume was associated with global brain atrophy measured by MRI (28). Furthermore, increased adiposity has been correlated with atrophy of several brain regions including the hippocampus (29, 30). Reduced hippocampal volume predicts cognitive function in the general population (31). Mid-life obesity was associated with earlier onset of Alzheimer's disease and greater severity of Alzheimer's neuropathology (4). Moreover, obesity increased the risk of cognitive impairment and dementia through vascular effects, such as atherosclerosis, endothelial dysfunction, arterial stiffness, and impairment of the blood-brain barrier (32, 33). Despite these definite direct and indirect links between mid-life obesity and dementia, epidemiological studies have focused on the association between late-life obesity and dementia and reported conflicting results. Weight reduction in midlife can lead to healthy condition via controlling various metabolic risk factors, whereas weight loss in late-life may reflect underlying illness and muscle reduction, causing unhealthy body composition. As a result, The association between a high Bwt and adverse clinical outcomes attenuates with age (10). Findings from the Whitehall II study published after the meta-analysis showed a higher risk of dementia for obesity at age 50, but not at ages 60–70 years (34). Furthermore, in the collaborative study of over 1.3 million adults, higher BMI was associated with increased dementia risk when weight was measured >20 years before dementia diagnosis, but this association was reversed when BMI was assessed <10 years before dementia diagnosis (35). Dementia-related weight loss appeared about 6 years before the onset of the clinical syndrome (36). Therefore, Bwt of elderly population cannot give accurate information for the risk of dementia.

Recently, several studies have demonstrated that Bwt variability was associated with increased risk of cardiovascular disease and mortality (12–14). According to the study in mice, weight cycling induced greater adipose tissue inflammation and insulin resistance than a consistently obese state (37) and a human study showed that Bwt fluctuation was related to poorer body-fat distribution (38). Sustained fluctuations in energy balance will lead to potential fluctuations of cardiometabolic risk variables, such as blood pressure, heart rate, blood glucose and lipids (39). In a 7-year follow-up French national cohort study, weight fluctuation was an independent risk factor for metabolic syndrome (40). Apart from this direct causal effect between Bwt variability and metabolic disturbances, Bwt fluctuation might reflect difficulty maintaining homeostasis, subsequent declining mental health. However, very few studies have assessed the effects of Bwt variability on dementia. One study suggested that mid-life Bwt variability was associated increased risk of dementia late in life (20). Another study of elderly women reported that Bwt variability was associated with increased risk of cognitive impairment or dementia, but the risk was attenuated after adjusting for covariates (21). However, those studies included a relative small-sized number with only women or men and did not utilize the accurate variability index such as CV, SD and VIM. Furthermore, the trajectory period was over 20-years covering both mid and late-life, which did not focus on the late-life Bwt variability like our research. We for the first time demonstrated that Bwt variability in the elderly was significantly associated with the increased risk for dementia after adjusting other covariates, suggesting that the inability to maintain one's weight might be an indication for poorer homeostatic control and general condition. This association remained statistical significance even after excluding the subject with continuous weight loss and very old age group (≥85 years), implying that the close relation between Bwt variability in late-life and dementia was independent from preclinical weight loss of dementia or age. Previously, Arnold et al. (41) reported that a history of weight cycling for the elderly with more than 65-years old increased the risk of physical disability by 28% after adjusting other risk factors including average weight and weight change per year. Considering that Bwt fluctuations in older persons are not benign condition, monitoring the weight of an older person for fluctuations as well as episodes of weight loss might be an important aspect of geriatric care.

Subgroup analyses in our study showed a stronger association between Bwt variability and dementia in people age <75 years compared with those older 75 years, because the older subgroup is easy to face with additional competing risk factors for dementia, such as age itself. The association between Bwt variability and dementia is stronger in subject with abnormal baseline BMI (underweight, overweight, and obese) compared to those with normal baseline BMI. Although the underlying mechanism of this association is not clear, individuals who exercised regularly had a higher risk of dementia according to Bwt variability quartile compared to those who did not (*P*-value for interaction = 0.048). Individuals without regular exercise might be more vulnerable to other risk factors of the dementia other than Bwt variability.

Our study has several limitations. We could not consider educational level or dietary factors due to lack of information. We could not consider the role of APOE e4, the major susceptibility allele for dementia, due to lack of data. Patients with dementia were identified according to ICD-10 codes and not confirmed by brain imaging. Thus, the prevalence of dementia may have been underestimated because we used information recorded in the claim database. The incidence of vascular dementia was relatively low, which may have reduced the significance of any associations. Finally, because of the intrinsic limitations of the observational study design, we could not define a causal relationship between Bwt variability and dementia. Despite these limitations, this study has the strength of using a large-scale nationwide database, which is standardized and validated by the Korean government and represents the entire elderly Korean population.

In conclusion, our study findings suggest that Bwt variability is an independent predictor for dementia in the elderly. The risk of developing dementia was significantly increased in subjects with highest Bwt variability and underweight BMI and significantly decreased in subjects with low Bwt variability and obese BMI. These results merit further study in order to confirm our results in different ethnic groups and to explore the mechanistic links between Bwt variability and dementia.

## Data Availability Statement

Data are available through the Korean National Health Insurance Sharing Service (NHISS). Researchers who wish to access the data can apply at (https://nhiss.nhis.or.kr/bd/ay/bdaya001iv.do) and request access to NHIS-2018–2-195.

## Ethics Statement

The studies involving human participants were reviewed and approved by Institutional Review Board of Korea University. Written informed consent for participation was not required for this study in accordance with the national legislation and the institutional requirements.

## Author Contributions

ER and HY contributed to the study design, statistical analyses, data interpretation, and fund raising and drafted the manuscript. SHw contributed to the data collection and statistical analyses. JK, Y-BL, and SHo contributed to the study design and data interpretation. NHoK, JS, SK, NHeK, KC, and SB contributed to the data interpretation. All authors have approved the submitted manuscript.

## Conflict of Interest

The authors declare that the research was conducted in the absence of any commercial or financial relationships that could be construed as a potential conflict of interest.
