# Supplementary material for: Body Weight Variability Increases Dementia Risk Among Older Adults: A Nationwide Population-Based Cohort Study
Source: Front Endocrinol (Lausanne). 2020 May 12;11:291. doi: 10.3389/fendo.2020.00291 (PMC7247844; doi:10.3389/fendo.2020.00291)
Supplement: Supplementary file 1 [file Data_Sheet_1.docx]

**Body weight variability and the risk of dementia among older adults: a nationwide population-based cohort study**

Eun Roh^1^, Soon Young Hwang^2^, Jung A Kim^1^, You-Bin Lee^1^, So-hyeon Hong^1^, Nam Hoon Kim^1^, Ji A Seo^1^, Sin Gon Kim^1^, Nan Hee Kim^1^, Kyung Mook Choi^1^, Sei Hyun Baik^1^, Hye Jin Yoo^1*^

^1^Division of Endocrinology and Metabolism, Department of Internal Medicine, Korea University College of Medicine, Seoul, Korea; ^2^Department of Biostatistics, Korea University College of Medicine, Seoul, Korea

**Supplementary Table 1.** Baseline characteristics of the subjects according to the presence of dementia

|  | All participants | No dementia | Dementia | *P*-value |
| --- | --- | --- | --- | --- |
|  | N = 19987 | N = 18395 | N = 1592 |  |
| Age (years) | 72(70,76) | 72(70,75) | 74(72,78) | <.001 |
| Sex (male) (n, %) | 12025(60.2) | 11232(61.1) | 793(49.8) | <.001 |
| BMI (kg/m^2^) | 23.51(21.5,25.46) | 23.53(21.55,25.47) | 23.07(21.05,25.15) | <.001 |
| Body weight (kg) | 59(52,66) | 59(53,66) | 56(50,63) | <.001 |
| Waist circumference (cm) | 83(78,89) | 83(78,89) | 82(77,88.5) | 0.001 |
| Body weight variability |  |  |  |  |
| VIM | 1.56(1.02,2.41) | 1.56(1.01,2.38) | 1.74(1.13,2.58) | <.001 |
| CV (%) | 2.66(1.71,4.08) | 2.63(1.69,4.03) | 3.09(1.95,4.65) | <.001 |
| SD (mg/dL) | 1.53(1,2.38) | 1.53(1,2.31) | 1.73(1.15,2.57) | <.001 |
| Systolic BP (mmHg) | 130(120,140) | 130(120,140) | 130(120,140) | 0.07 |
| Diastolic BP (mmHg) | 80(70,84) | 80(70,84) | 80(70,83) | 0.011 |
| Fasting plasma glucose (mg/dL) | 97(88,109) | 97(88,108) | 97(88,110) | 0.748 |
| Total cholesterol (mg/dL) | 194(171,220) | 194(171,220) | 194(171,223) | 0.44 |
| Aspartate transaminase (IU/L) | 24(20,29) | 24(20,29) | 24(20,29) | 0.104 |
| Alanine transaminase (IU/L) | 19(15,25) | 19(15,26) | 18(14,24) | <.001 |
| Current smoker (n, %) | 2988(14.9) | 2795(15.2) | 193(12.1) | 0.001 |
| Alcohol consumption (n, %) | 6399(32) | 5978(32.5) | 421(26.4) | <.001 |
| Regular exercise (n, %) | 3935(19.7) | 3649(19.8) | 286(18) | 0.072 |
| Diabetes (n, %) | 4958(24.8) | 4463(24.3) | 495(31.1) | <.001 |
| Hypertension (n, %) | 12790(64) | 11737(63.8) | 1053(66.1) | 0.062 |
| Dyslipidemia (n, %) | 6625(33.1) | 6038(32.8) | 587(36.9) | 0.001 |
| Cerebrovascular disease (n, %) | 1762(8.8) | 1530(8.3) | 232(14.6) | <.001 |
| Ischemic heart disease (n, %) | 2325(11.6) | 2113(11.5) | 212(13.3) | 0.029 |
| Income (lower 10%) | 2231(11.2) | 2062(11.2) | 169(10.6) | 0.470 |
| *P* value derived using Mann-Whitney test.  Data are expressed as median (interquartile range), or n (%).  Abbreviation: BMI, body mass index; BP, blood pressure; CV, coefficients of variance; SD, standard deviation; VIM, variability independent of the mean | | | | |

**Supplementary Table 2.** Baseline characteristics of the subjects according to the baseline BMI

|  | < 18 kg/m^2^  (underweight) | 18 – 22.9 kg/m^2^ (normal weight) | 23 – 24.9 kg/m^2^ (overweight) | > 25 kg/m^2^ (obese) | *P*-value |
| --- | --- | --- | --- | --- | --- |
|  | N = 538 | N =8120 | N = 5282 | N = 6047 |  |
| Age (years) | 74(70,78) | 72(70,76) | 72(70,74) | 72(70,74) | <.001 |
| Sex (male) (n, %) | 330(61.3) | 5155(63.5) | 3316(62.8) | 3224(53.3) | <.001 |
| BMI (kg/m^2^) | 17.19(16.56,17.6) | 21.33(20.2,22.21) | 23.96(23.51,24.46) | 26.57(25.68,27.92) | <.001 |
| Body weight (kg) | 43(39,47) | 53(48,58) | 61(56,66) | 67(61,73) | <.001 |
| Waist circumference (cm) | 69(65,73) | 78(74,82) | 84(81,88) | 90(86,94) | <.001 |
| Body weight variability |  |  |  |  |  |
| VIM | 1.84(1.2,3.14) | 1.59(1.04,2.46) | 1.53(1,2.28) | 1.53(1,2.41) | <.001 |
| CV (%) | 4.07(2.53,6.69) | 2.99(1.92,4.47) | 2.52(1.63,3.76) | 2.37(1.56,3.63) | <.001 |
| SD (mg/dL) | 1.73(1.15,3) | 1.53(1,2.45) | 1.53(1,2.31) | 1.53(1,2.52) | <.001 |
| Incidence of dementia (n, %) |  |  |  |  |  |
| All-cause dementia | 63(11.7) | 724(8.9) | 384(7.3) | 421(7) | <.001 |
| Vascular dementia | 12(2.2) | 147(1.8) | 66(1.3) | 79(1.3) | 0.013 |
| Alzheimer’s dementia | 48(8.9) | 546(6.7) | 304(5.8) | 319(5.3) | <.001 |
| Systolic BP (mmHg) | 120(110,135) | 130(119,139) | 130(120,140) | 130(120,140) | <.001 |
| Diastolic BP (mmHg) | 77(70,80) | 80(70,82) | 80(70,84) | 80(71,85) | <.001 |
| Fasting plasma glucose (mg/dL) | 94(86,103) | 95(87,105) | 97(89,109) | 99(91,113) | <.001 |
| Total cholesterol (mg/dL) | 187(164,209) | 192(169,216) | 195(172,221) | 198(173,225) | <.001 |
| Aspartate transaminase (IU/L) | 25(21,31) | 24(20,29) | 24(20,29) | 24(20,30) | <.001 |
| Alanine transaminase (IU/L) | 17(13,21) | 18(14,23) | 20(15,26) | 21(16,29) | <.001 |
| Current smoker (n, %) | 126(23.4) | 1561(19.2) | 686(13) | 615(10.2) | <.001 |
| Alcohol consumption (n, %) | 156(29) | 2718(33.5) | 1737(32.9) | 1788(29.6) | <.001 |
| Regular exercise (n, %) | 79(14.7) | 1564(19.3) | 1126(21.3) | 1166(19.3) | <.001 |
| Diabetes (n, %) | 91(16.9) | 1597(19.7) | 1361(25.8) | 1909(31.6) | <.001 |
| Hypertension (n, %) | 233(43.3) | 4461(54.9) | 3503(66.3) | 4593(76) | <.001 |
| Dyslipidemia (n, %) | 97(18) | 2186(26.9) | 1819(34.4) | 2523(41.7) | <.001 |
| Cerebrovascular disease (n, %) | 50(9.3) | 599(7.4) | 508(9.6) | 605(10) | <.001 |
| Ischemic heart disease (n, %) | 43(8) | 786(9.7) | 588(11.1) | 908(15) | <.001 |
| Income (lower 10%) | 62(11.5) | 944(11.6) | 568(10.8) | 657(10.9) | 0.354 |
| *P* value derived using Kruskal-Wallis test and chi-square tests.  Data are expressed as median (interquartile range), or n (%).  Abbreviation: BMI, body mass index; BP, blood pressure; CV, coefficients of variance; SD, standard deviation; VIM, variability independent of the mean | | | | | |

**Supplementary Table 3.** Adjusted hazard ratios and 95% confidence intervals for the incidence of dementia according to groups categorized by baseline body mass index (BMI) and body weight (Bwt) variability

| Bwt variability  Baseline BMI | Q1 | Q2 | Q3 | Q4 | *P* for interaction |
| --- | --- | --- | --- | --- | --- |
| BMI < 18 kg/m^2^ (underweight) | 0.87 (0.458,1.651) | 1.245 (0.72,2.155) | 1.247 (0.721,2.159) | 1.994 (1.302,3.054) | 0.0337 |
| BMI 18 – 22.9 kg/m^2^ (normal weight) | 1 (ref) | 1.182 (0.953,1.467) | 1.174 (0.947,1.454) | 1.151 (0.93,1.424) |  |
| BMI 23 – 24.9 kg/m^2^ (overweight) | 0.867 (0.662,1.134) | 0.711 (0.538,0.939) | 0.997 (0.776,1.281) | 1.113 (0.875,1.416) |  |
| BMI > 25 kg/m^2^ (obese) | 0.664 (0.505,0.872) | 0.648 (0.493,0.852) | 0.949 (0.744,1.211) | 1.108 (0.879,1.397) |  |
| Adjusted for age, sex, alcohol, smoking, exercise, income, diabetes, hypertension, dyslipidemia, ischemic heart disease, cerebrovascular disease | | | | | |

**Supplementary Table 4.** Hazard ratios and 95% confidence intervals for the incidence of dementia by quartiles of body weight (Bwt) variability after excluding subjects aged 85 years and older

|  | Total (n) | Events (n) | Follow-up duration (person years) | Incidence rate  (per 1000 person years) | Hazard ratio (95% confidence intervals) | | | |
| --- | --- | --- | --- | --- | --- | --- | --- | --- |
|  |  |  |  |  | Unadjusted | Model 1 | Model 2 | Model 3 |
| **All-cause dementia** | | | | | | | | |
| Q1 | 4949 | 312 | 31505.16 | 9.90 | 1 (ref) | 1 (ref) | 1 (ref) | 1 (ref) |
| Q2 | 4932 | 341 | 31396.8 | 10.86 | 1.096 (0.94,1.278) | 1.069 (0.917,1.247) | 1.061 (0.91,1.237) | 1.063 (0.912,1.24) |
| Q3 | 4952 | 425 | 31242.34 | 13.60 | 1.377 (1.19,1.593) | 1.301 (1.124,1.505) | 1.275 (1.101,1.477) | 1.271 (1.097,1.472) |
| Q4 | 4900 | 484 | 30766.9 | 15.73 | 1.593 (1.382,1.837) | 1.451 (1.258,1.674) | 1.432 (1.242,1.653) | 1.426 (1.236,1.646) |
| *P* for trend | |  |  |  | <.001 | <.001 | <.001 | <.001 |
| **Vascular dementia** | | | | | | | | |
| Q1 | 4949 | 69 | 32130.98 | 2.15 | 1 (ref) | 1 (ref) | 1 (ref) | 1 (ref) |
| Q2 | 4932 | 64 | 32073.12 | 2.00 | 0.929 (0.661,1.305) | 0.911 (0.648,1.28) | 0.902 (0.642,1.268) | 0.902 (0.642,1.268) |
| Q3 | 4952 | 74 | 32138.55 | 2.30 | 1.073 (0.773,1.49) | 1.026 (0.739,1.425) | 1.003 (0.721,1.395) | 0.984 (0.707,1.368) |
| Q4 | 4900 | 92 | 31752.33 | 2.90 | 1.351 (0.989,1.846) | 1.255 (0.917,1.716) | 1.236 (0.903,1.692) | 1.198 (0.875,1.64) |
| *P* for trend | |  |  |  | 0.039 | 0.116 | 0.146 | 0.219 |
| **Alzheimer’s dementia** | | | | | | | | |
| Q1 | 4949 | 226 | 31767.41 | 7.11 | 1 (ref) | 1 (ref) | 1 (ref) | 1 (ref) |
| Q2 | 4932 | 261 | 31618.33 | 8.25 | 1.159 (0.97,1.385) | 1.131 (0.947,1.352) | 1.122 (0.939,1.341) | 1.126 (0.942,1.346) |
| Q3 | 4952 | 332 | 31544 | 10.52 | 1.483 (1.252,1.756) | 1.399 (1.181,1.656) | 1.369 (1.156,1.623) | 1.372 (1.158,1.626) |
| Q4 | 4900 | 373 | 31136.39 | 11.98 | 1.686 (1.43,1.989) | 1.53 (1.296,1.806) | 1.509 (1.278,1.782) | 1.513 (1.281,1.787) |
| *P* for trend | |  |  |  | <.001 | <.001 | <.001 | <.001 |
| Model 1: Adjusted for age, sex  Model 2: model 1 + BMI  Model 3: model 2 + alcohol, smoking, exercise, income, diabetes, hypertension, dyslipidemia, ischemic heart disease, cerebrovascular disease | | | | | | | | |

**Supplementary Table 5.** Hazard ratios and 95% confidence intervals of incidence dementia by quartiles of body weight (Bwt) variability after excluding subjects with continuous weight loss or gain

|  | Total (n) | Events (n) | Follow-up duration (person years) | Incidence rate  (per 1000 person years) | Hazard ratio (95% confidence intervals) | | | |
| --- | --- | --- | --- | --- | --- | --- | --- | --- |
|  |  |  |  |  | Unadjusted | Model 1 | Model 2 | Model 3 |
| **All-cause dementia** | | | | | | | | |
| Q1 | 4495 | 292 | 28587.08 | 10.21 | 1 (ref) | 1 (ref) | 1 (ref) | 1 (ref) |
| Q2 | 4023 | 287 | 25558.39 | 11.23 | 1.099(0.934,1.294) | 1.082(0.919,1.273) | 1.075(0.914,1.266) | 1.074(0.913,1.265) |
| Q3 | 3832 | 319 | 24217.99 | 13.17 | 1.291(1.102,1.513) | 1.237(1.055,1.45) | 1.218(1.039,1.428) | 1.212(1.033,1.421) |
| Q4 | 3192 | 307 | 20079.58 | 15.29 | 1.499(1.277,1.76) | 1.35(1.15,1.586) | 1.338(1.139,1.572) | 1.325(1.127,1.557) |
| *P* for trend | |  |  |  | <.001 | <.001 | <.001 | <.001 |
| **Vascular dementia** | | | | | | | | |
| Q1 | 4495 | 62 | 29171.32 | 2.13 | 1 (ref) | 1 (ref) | 1 (ref) | 1 (ref) |
| Q2 | 4023 | 53 | 26134.55 | 2.03 | 0.954(0.661,1.376) | 0.941(0.652,1.358) | 0.935(0.648,1.349) | 0.928(0.643,1.339) |
| Q3 | 3832 | 56 | 24880.19 | 2.25 | 1.06(0.739,1.522) | 1.025(0.714,1.472) | 1.008(0.701,1.447) | 0.984(0.685,1.415) |
| Q4 | 3192 | 59 | 20699.67 | 2.85 | 1.343(0.941,1.919) | 1.242(0.868,1.777) | 1.23(0.859,1.76) | 1.186(0.828,1.698) |
| *P* for trend | |  |  |  | 0.086 | 0.205 | 0.233 | 0.327 |
| **Alzheimer’s dementia** | | | | | | | | |
| Q1 | 4495 | 215 | 28816.76 | 7.46 | 1 (ref) | 1 (ref) | 1 (ref) | 1 (ref) |
| Q2 | 4023 | 222 | 25748.75 | 8.62 | 1.155(0.958,1.394) | 1.135(0.941,1.369) | 1.128(0.935,1.361) | 1.129(0.936,1.363) |
| Q3 | 3832 | 247 | 24448.1 | 10.10 | 1.356(1.129,1.628) | 1.296(1.08,1.556) | 1.275(1.061,1.531) | 1.275(1.061,1.532) |
| Q4 | 3192 | 232 | 20322.98 | 11.42 | 1.529(1.27,1.841) | 1.368(1.135,1.648) | 1.354(1.123,1.632) | 1.352(1.122,1.63) |
| *P* for trend | |  |  |  | <.001 | <.001 | 0.001 | 0.001 |
| Model 1: Adjusted for age, sex  Model 2: model 1 + BMI  Model 3: model 2 + alcohol, smoking, exercise, income, diabetes, hypertension, dyslipidemia, ischemic heart disease, cerebrovascular disease | | | | | | | | |
